# Contrasting development of lysigenous aerenchyma in two rice genotypes under phosphorus deficiency

**DOI:** 10.1186/s13104-018-3179-y

**Published:** 2018-01-22

**Authors:** Vincent Pujol, Matthias Wissuwa

**Affiliations:** Crop, Livestock and Environment Division, Japan International Research Center for Agricultural Sciences (JIRCAS), Tsukuba, Ibaraki Japan

**Keywords:** Lysigenous aerenchyma, Phosphorus deficiency, Rice, Root porosity, Phosphorus use efficiency

## Abstract

**Objectives:**

Phosphorus (P) deficiency is a major limitation to plant growth. Under several abiotic stresses, including P deficiency, upland cereal crops, such as maize, are well known to develop lysigenous aerenchyma, a root tissue containing gas spaces. Contrary to upland species, rice develops aerenchyma constitutively. Nevertheless, aerenchyma in rice is also enhanced by several abiotic stresses, including P deficiency. However, studies are limited and genotypic differences are not clear.

**Results:**

The formation of inducible aerenchyma in response to P deficiency was evaluated in two rice genotypes, DJ123 and Nerica4. Whole root porosity increased for both genotypes in low P conditions, but was more pronounced in DJ123. Direct aerenchyma measurements, at 20 and 30 mm from the seminal root tip, revealed that aerenchyma in low P conditions was only enhanced in DJ123. These results confirm that P deficiency in rice induces the formation of aerenchyma, and further show that genotypic differences exist. Interestingly, DJ123 is considered tolerant to P deficiency, whereas Nerica4 is sensitive, pointing towards a potential role of aerenchyma in tolerance to P deficiency.

**Electronic supplementary material:**

The online version of this article (10.1186/s13104-018-3179-y) contains supplementary material, which is available to authorized users.

## Introduction

Phosphorus (P) deficiency is a major limitation to plant growth and is typically counterbalanced through the heavy application of mineral fertilizers, which is inefficient, polluting and expensive [1, 2]. Improving plants’ ability to take up and use P efficiently is therefore essential for the development of sustainable farming and to ensure global food security. In this regard, lysigenous aerenchyma is seen as a positive factor, as it allows the plant to reduce cell cost maintenance as well as to remobilize nutrients, thereby enabling growth [3, 4].

Aerenchyma is a tissue containing enlarged gas spaces, which allow the diffusion of gases, notably, oxygen from shoots to roots, and CO_2_ and ethylene from roots to shoots [5]. In cereal crops such as rice, maize, barley and wheat, lysigenous aerenchyma is formed in the root cortex following the death of cortical cells, via programmed cell death [5]. In dryland plants, lysigenous aerenchyma is known to be induced by waterlogging and nutrient deficiency [5–7]. In contrast, rice (*Oryza sativa* L.) forms lysigenous aerenchyma constitutively, without experiencing any stress [8]. Nevertheless, aerenchyma is also enhanced by several abiotic stresses [9–11], including P deficiency [12]. This was mainly observed via the increase in root porosity, which indirectly reflects the formation of aerenchyma [13–15]. To date, genotypic differences are not clear and the role of aerenchyma in tolerance to P deficiency has not been addressed.

In this study, the formation of inducible lysigenous aerenchyma in rice, in response to low P conditions, was evaluated in detail to complement previous studies. Two rice genotypes, DJ123 (DJ) and Nerica4 (N4), were used to detect potential genotypic differences. In addition, these genotypes contrast in their tolerance to P deficiency; N4 is sensitive while DJ is tolerant [16, 17]. Seedlings were grown under high and low P conditions and whole root porosity as well as the proportion of aerenchyma near the seminal root tip were evaluated.

## Main text

### Methods

#### Plant growth

Two rice varieties were used: DJ123 (DJ–*O. sativa ssp. aus*) and Nerica4 (N4–*O. sativa* × *O. glaberrima*). Seeds of DJ123 were sourced from the IRRI gene bank (IRGC117711) and seeds of Nerica4 were held at JIRCAS after an original import from Africa in 2010. Seeds were incubated at 50 °C for 2 days to break dormancy, then surface-sterilized (0.5% NaOCl + 0.15 M KH_2_PO_4_) and germinated in the dark at 28 °C for 3 days. Uniform germinated seeds were transferred onto floating mesh in 20 L deionized water in glasshouse (light > 12 h, 26–30 °C, humidity 50–90%). Genotypes were grown together in two (microscopy 1) or three (porosity and microscopy 2) containers per P treatment. FeEDTA (12 µM) and CaCl_2_ (100 µM) were added after 2 days. From 10 days after germination, solution was replaced on a weekly basis with modified Yoshida solution [18] at 1/3, 1/2 and full strength. Full strength Yoshida solution contained NH_4_NO_3_ (N: 2.86 mM), K_2_SO_4_ (K: 1.02 mM), CaCl_2_·2H_2_O (Ca: 1 mM), MgSO_4_·7H_2_O (Mg: 1.65 mM), MnCl_2_·4H_2_O (Mn: 9.1 µM), (NH_4_)_6_·Mo_7_O_24_·4H_2_O (Mo: 0.52 µM), H_3_BO_3_ (B: 18.5 µM), ZnSO_4_·7H_2_O (Zn: 0.15 µM), CuSO_4_·5H_2_O (Cu: 0.16 µM) and EDTA iron(III) sodium salt (Fe: 35.81 µM). P treatment (NaH_2_PO_4_·2H_2_O) started 10 days after germination, in quantities corresponding to 100 (high) and 1 (low) µM P in full strength Yoshida solution. pH was regularly adjusted to approx. 5.7 and the solution was constantly aerated by gentle bubbling.

#### Root porosity

At 17 and 18 days post-treatment (DPT), six replicates (three per day), corresponding to three pooled plants (one from each container), were sampled in distilled water and whole root porosity was quickly measured using a pycnometer method [19, 20]. Fifty-milliliter pycnometers (one for each sample) were filled with distilled water and weighed (PW) on an analytical balance with 0.1 mg accuracy (Mettler Toledo AG204). Roots were transferred into each pycnometer and weighed (PWR). Water excess on roots was removed by centrifugation and root fresh weight was quickly measured (R); roots were kept in a sealed container to prevent drying. Roots were cut into approx. 1 cm long segments, transferred to distilled water and air was removed by a series of vacuuming (approx. 30 min total). Infiltrated root segments were then transferred back into the same pycnometers and weighed one last time (PWRi). Pycnometers and distilled water were always kept at constant temperature and handled with minimal contact to avoid temperature change. Nevertheless, water temperature was immediately measured after each weighing, using an analogue thermometer (± 0.25 °C) and water weight was adjusted accordingly. Porosity was calculated after water weight normalization using the formula: %Porosity = 100 × (PWRi − PWR)/(PW − PWR + R).

#### Proportion of aerenchyma

For the first experiment, eight replicates (four from each container) were sampled at 15 DPT. For the second experiment, seedlings were sampled at 7 and 14 DPT in six and nine replications (three per container) from high and low P conditions, respectively; one high P container was discarded as plants were dissimilar from the others. Root length was measured with a ruler (± 0.5 cm).

For each sample, the seminal root was sampled in Yoshida solution and 5 mm root segments around 20 and 30 mm from the root tip were cut, transferred in Yoshida solution and vacuumed for approx. 20 min to remove air bubbles. Segments were transferred into 5 ml 3% agarose (NE-AG02 Fast Gene) at 50 °C. Once solidified, blocks were sealed with plastic wrap to avoid drying and stored at 4 °C until processing (up to 9 days, no alterations observed). 75 µm-sections were prepared from the middle of the root segment with a vibratome (DSK Microslicer DTK-1000) and platinum coated double edge blades (Electron Microscopy Sciences). Cross sections were stained for 30 s in 0.0005% Toluidine blue and photographed using bright field microscopy (Olympus BX50) at 10× with an Olympus DP21 camera. For each segment, the best five pictures were manually analyzed in ImageJ (v1.50g) and root, cortex, stele and aerenchyma area were measured. Cortex zones that were damaged or adjacent to lateral roots, where aerenchyma does not develop, were excluded from measurements. Total aerenchyma area was normalized according to the percent of measured cortex. The mean of all five pictures was used for analysis.

#### Statistical analysis

Statistical analyses were performed in R (v3.3.0) [21, 22]. Statistical differences between the means of biological replicates were evaluated using two-way ANOVA, with genotype and treatment as factors, followed by Tukey’s HSD post hoc tests. Mean differences were considered statistically significant at *p* value ≤ 0.05.

### Results

**Fig. 1:**
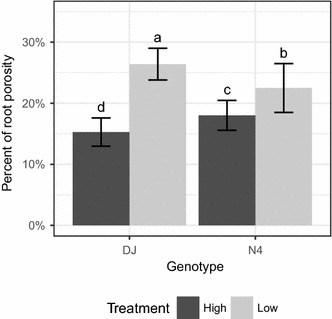
Root porosity. Means with standard deviation (n = 6 biological replicates, each corresponding to three pooled plants) of whole root porosity for each genotype grown in high (black) and low (grey) P conditions. Plants were sampled at 17 and 18 DPT and results pooled together. Means with different letters are significantly different (ANOVA followed by Tukey’s HSD at p ≤ 0.05)

**Fig. 2:**
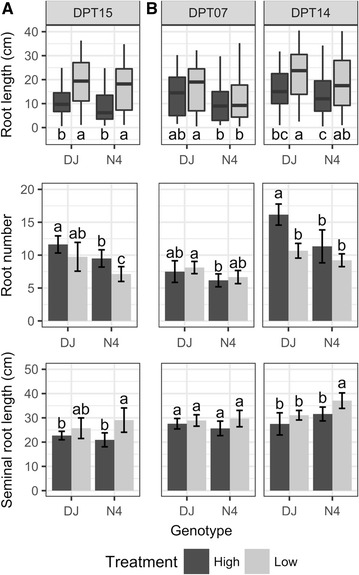
Root length and number. Root length distribution of all roots and all plants (top panels) and means with standard deviation (n = 6 to 9 biological replicates) of root number (middle panels) and seminal root length (bottom panels), for each genotype grown in high (black) and low (grey) P conditions,. The lower and upper hinge of the box show the 25th and 75th percentiles, respectively, the middle line shows the median and the whiskers show the minimum and maximum values. Plants were sampled (**A**) at 15 DPT (left panels) for the first experiment and (**B**) at 07 (middle panels) and 14 (right panels) DPT for the second experiment. Means with different letters are significantly different (ANOVA followed by Tukey’s HSD at p ≤ 0.05)

Root porosity increased for both genotypes in low P (Fig. 1) and was more pronounced in DJ (+73%) than in N4 (+23%). In the microscopy experiments, roots were in some cases less numerous in low P than in high P at 14 and 15 DPT, but not at 7 DPT (Fig. 2). The length of all roots was very similar between treatments at 7 DPT, whereas at later time points, most roots were clearly longer in low P than in high P (Fig. 2). However, DJ’s seminal root was not significantly longer in low P, as opposed to N4 (Fig. 2). Interestingly, DJ’s seminal root, in both treatments, did not grow much from 7 to 14 DPT, whereas for N4, it grew by 23% (two-tailed t Test: t = − 3.5, df = 10, p = 0.006) and 25% (t = − 4.8, df = 16, p = 0.0002) in high and low P, respectively. Root diameter at 20 and 30 mm from DJ’s seminal root tip was always similar between treatments (Additional file 1), but was wider in N4 in the second experiment; nevertheless, the proportion of aerenchyma in the cortex remained comparable.

**Fig. 3:**
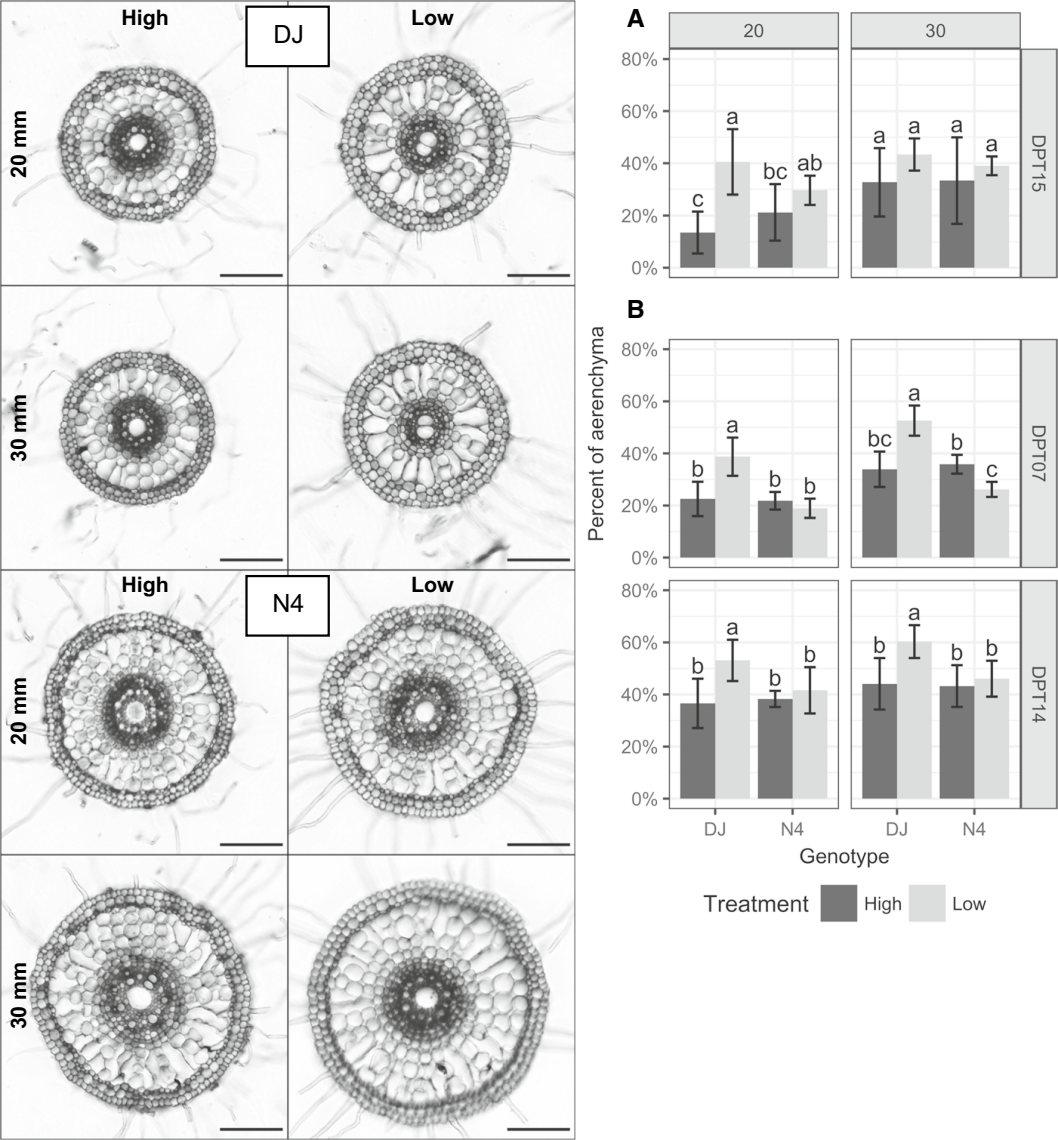
Proportion of aerenchyma. Left: cross-sections at 20 and 30 mm from root tip of seminal root of each genotype grown in high and low P conditions. Pictures were taken in bright field microscopy and correspond to the average development of aerenchyma for each genotype and each treatment at 07 DPT. Scale bars represent 100 µm. Right: means with standard deviation (n = 4 to 9 biological replicates) of the proportion of aerenchyma in seminal roots at 20 (left panels) and 30 (right panels) mm from the root tip of plants grown in high (black) and low (grey) P conditions. Plants were sampled (**A**) at 15 DPT for the first experiment and (**B**) at 07 and 14 DPT for the second experiment. Means with different letters are significantly different (ANOVA followed by Tukey’s HSD at p ≤ 0.05)

Except in the first experiment at 30 mm from the root tip (Fig. 3A), for which results were not statistically significant, the proportion of aerenchyma in DJ was always higher in low P than in high P (Fig. 3). At 20 mm, between 39 and 53% of the cortex was converted to aerenchyma in low P, corresponding to an increase of 45–200% in response to P deficiency. At 30 mm, the proportion of aerenchyma was between 53 and 60%, corresponding to an increase of 37–55%. At that position (30 mm), most of the aerenchyma was formed; the proportion cannot reach 100% due to remaining cell walls. In N4, the proportions of aerenchyma did not vary between treatments and were similar to typical proportions seen in DJ in high P. For both genotypes in the second experiment (Fig. 3B), the proportion of aerenchyma was higher at 14 DPT than at 7 DPT, independently of the treatment or the location in the root, indicating an increase in the formation of constitutive aerenchyma over time.

### Discussion

Whole root porosity is known to correlate with aerenchyma formation [23]; as aerenchyma forms, porosity increases. Moreover, P deficiency is known to enhance root porosity in several species, including rice [13–15]. Here, a general increase in porosity in response to P deficiency was confirmed. Furthermore a pronounced difference in the increase of porosity between genotypes was found, with DJ displaying a far stronger response than N4. These results suggest that the formation of aerenchyma was enhanced by P deficiency.

To confirm these results, the proportion of aerenchyma was directly measured in the seminal root near the tip. As aerenchyma forms constitutively in rice roots, and progresses from the apical part to the basal part [24], it is important to study it at an early stage of development. Here, 20 and 30 mm from the root tip were found to be optimal, as aerenchyma was sometimes barely present at 20 mm, whereas in some cases, it was fully formed at 30 mm. Beyond this point, the formation of constitutive aerenchyma was expected to be complete, regardless of the genotype or treatment. In addition, enhancement of inducible aerenchyma was greater at 20 mm than at 30 mm, probably due to the lower proportion of constitutive aerenchyma.

In two independent experiments, P deficiency enhanced the proportion of aerenchyma in DJ, demonstrating that P deficiency induces the formation of aerenchyma. This is in agreement with Vejchasarn et al. [12]. However, such a response was not observed for N4. It can be ruled out that these results were an artifact caused by differences in tissue age of more rapidly elongating roots under P deficiency, as seminal roots at 7 DPT were similar in both treatments. The lack of enhanced aerenchyma in N4 contradicts the porosity results. However, as pointed out by Justin and Armstrong [25], root length is an important factor influencing root porosity: the longer the root, the higher the porosity (aerenchyma does not develop, or develops less in the apical part). In addition, other root structures promoted by P deficiency could affect root porosity. Both genotypes developed longer roots in low P and possibly fewer roots. Therefore, the increase in N4 porosity in response to P deficiency, which is much lower than that of DJ, was likely an artifact of longer roots.

Interestingly, N4 is sensitive to P deficiency while DJ is tolerant [16, 17]. This was confirmed in the field (Additional file 2: Table S2). Although the present study does not allow a direct link, one may speculate that the inducible aerenchyma observed in DJ increases its tolerance to P deficiency. Aerenchyma has the potential to improve P utilization efficiency (PUE) by reducing cell cost maintenance and recycling nutrients [3, 4]. In addition to utilizing P more efficiently, a genotype developing inducible aerenchyma would have the opportunity to develop additional root structures (e.g. longer roots, lateral roots and root hairs), which would then increase the volume of soil explored and thus increase P uptake. Interestingly, DJ has been shown to possess high internal PUE in root and shoot tissues [17] and carries a rare haplotype associated with increased PUE located on chromosome 11 [26]. DJ is also known to develop longer root hairs in P deficient conditions while N4 does not [27]. DJ’s inducible aerenchyma could be one component leading to higher PUE and possibly to longer root hairs. Such a trait could therefore be beneficial in breeding programs to develop rice varieties with tolerance to P deficiency.

## Limitations

Only two contrasting genotypes were used. More are needed to investigate the role of inducible aerenchyma in tolerance to P deficiency.

## Additional files

**Additional file 1.** Root, cortex and stele diameter.

**Additional file 2.** Plant biomass in hydroponics (Table 1) and plant phenotypes in the field (Table 2).

## Abbreviations

DJ: DJ123
DPT: days post-treatment
N4: Nerica4
P: phosphorus
