# Supplementary material for: Contrasting development of lysigenous aerenchyma in two rice genotypes under phosphorus deficiency
Source: BMC Res Notes. 2018 Jan 22;11:60. doi: 10.1186/s13104-018-3179-y (PMC5778689; doi:10.1186/s13104-018-3179-y)
Supplement: Supplementary file 1 — Additional file 1. Root, cortex and stele diameter. [file 13104_2018_3179_MOESM1_ESM.pdf]

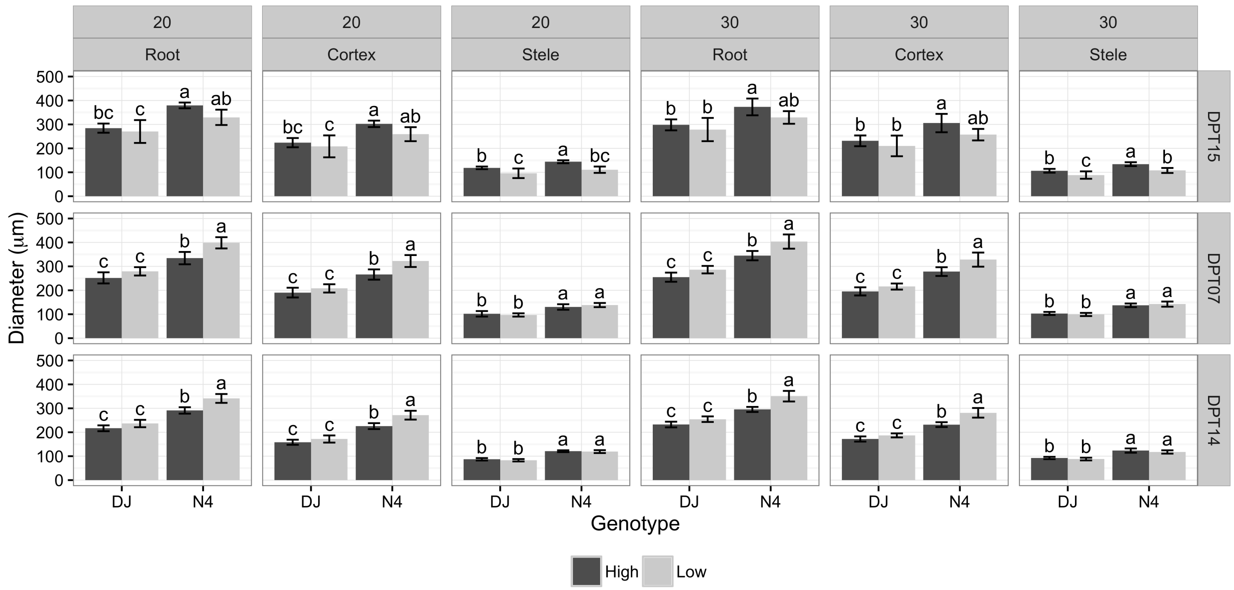

Root, cortex and stele diameter. Means with standard deviation (n = 4 to 9) of root, cortex and stele diameter in seminal roots at 20 (left three panels) and 30 (right three panels) mm from the root tip of plants grown in high (black) and low (grey) P conditions. Plants were sampled at 15 DPT for the first experiment (top panel) and at 07 and 14 DPT for the second experiment (bottom two panels). Means with different letters are significantly different (Tukey's HSD, p-value ≤ 0.05).
